# Cryptic transmission of a SARS-CoV-2 variant detected by wastewater surveillance in Panama

**DOI:** 10.3389/fcimb.2024.1467484

**Published:** 2025-01-29

**Authors:** Melissa Gaitán, Yamitzel Zaldivar, Michelle Hernandez, Jessica Góndola, Oris Chavarría, Brechla Moreno, Danilo Franco, Rodrigo DeAntonio, Santiago Mirazo, Florencia Cancela, Maria Eugenia Barnett, Alexander A. Martinez, Juan Miguel Pascale, Sandra López-Vergès

**Affiliations:** ^1^ Department of Research in Virology and Biotechnology, Gorgas Memorial Institute for Health Studies, Panama City, Panama; ^2^ Department of Research and Surveillance of Biologic Risk 3, Gorgas Memorial Institute for Health Studies, Panama City, Panama; ^3^ Department of Genomics and Proteomics, Gorgas Memorial Institute for Health Studies, Panama City, Panama; ^4^ Centro de Vacunacion e Investigacion CEVAXIN, Panama City, Panama; ^5^ Unidad Académica de Bacteriología y Virología, Instituto de Higiene, Facultad de Medicina, Universidad de la República, Montevideo, Uruguay; ^6^ Clinical Research Unit, Gorgas Memorial Institute for Health Studies, Panama City, Panama

**Keywords:** SARS-CoV-2, genomic surveillance, wastewater surveillance, outbreak, variant, molecular detection, complete genomes

## Abstract

The COVID-19 pandemic highlighted the critical role of viral genomic surveillance, prompting numerous countries to enhance their monitoring systems for acute respiratory infections (ARIs), especially influenza-like illnesses (ILIs). Given the significance of asymptomatic cases in severe acute respiratory syndrome coronavirus 2 (SARS-CoV-2) transmission, cases often undetected by the ILI surveillance, a more comprehensive approach was essential to track the circulation of SARS-CoV-2 variants in the population. In response, many countries swiftly adopted wastewater surveillance, which allowed the early detection of SARS-CoV-2 variants before they were identified through molecular characterization from confirmed clinical cases. In this report, we detail the implementation of SARS-CoV-2 wastewater genomic surveillance in Panama during the first half of 2024. Wastewater samples were collected monthly in duplicate at two collection points from three districts of Panama city metropolitan area for testing by SARS-CoV-2 RT-qPCR, and positive samples were analyzed by next-generation sequencing to identify sublineages. A total of 36 wastewater samples and 822 samples obtained through the clinical surveillance were analyzed for molecular detection and sequencing. Sublineages detected by wastewater surveillance were compared to those detected by clinical surveillance for the same period of time. Wastewater surveillance allowed the identification of the Omicron sublineage JN.1.16.1 in the capital city and its surroundings, which was not detected by the clinical surveillance in the country, despite its global circulation. This highlights the critical need to sustain both genomic surveillance programs beyond the pandemic in countries like Panama that serve as pivotal exchange hubs.

## 1 Introduction

Severe acute respiratory syndrome coronavirus 2 (SARS-CoV-2) was first detected in December 2019 in Wuhan city, Hubei province, China (Wang W. et al., 2020). In Panama, the first confirmed case of coronavirus disease 2019 (COVID-19) was reported on 9 March 2020 (Franco et al., 2021). The aggressiveness of the virus, its rapid spread, and the lack of knowledge about its evolution and behavior in individuals led the World Health Organization (WHO) to declare the new virus a pandemic on 11 March 2020 (Cucinotta and Vanelli, 2020; Wang W. et al., 2020). SARS-CoV-2, an RNA genome virus, belongs to the large family of coronaviruses. Coronaviruses infect animals, and seven of them can cause disease in humans, the most recent being SARS-CoV-2 (Wang L. et al., 2020).

The COVID-19 pandemic underlined the importance of molecular diagnosis through RT-PCR and of genomic surveillance for characterizing and monitoring SARS-CoV-2 variant circulation. The genomic surveillance of SARS-CoV-2 in human samples was based on the severe acute respiratory infections (SARIs) and influenza-like illness (ILI) existing molecular surveillance (Denayer et al., 2023; Marcenac et al., 2022); however, the importance of asymptomatic patients in SARS-CoV-2 transmission (Ferretti et al., 2020; Mizumoto et al., 2020) induced the inclusion of these nasopharyngeal swab specimens in the molecular surveillance and even the genomic surveillance. The regional genomic surveillance of SARS-CoV-2 in Latin America, a collaboration between countries under Pan-American Health Organization (PAHO) guidance, has been focused on characterizing the virus from symptomatic patients and asymptomatic individuals (Gräf et al., 2024). However, with the end of the pandemic, mass vaccination, and the decline in severe symptomatic cases, human surveillance has greatly diminished, making more difficult the analysis of circulating variants and early detection of variants of interest, such as those that escape existing vaccines.

Several studies have shown that SARS-CoV-2 RNA is present in the stool of patients with COVID-19 regardless of the presence of gastrointestinal symptoms or the severity of the disease (Chen et al., 2020). It has also been shown that the virus can be excreted in the feces for long periods of time during illness and convalescence, with several studies detecting it up to several weeks (between 1 and 5) after negativization in respiratory specimens (Xu et al., 2020). SARS-CoV-2 has also been detected in feces of asymptomatic people (Tang et al., 2020). Several research groups were able to isolate infectious SARS-CoV-2 from stool samples of patients with COVID-19 (Wang W. et al., 2020; Zhang et al., 2020), demonstrating that the virus can replicate in the intestinal tract (Zhou et al., 2020), even if other groups were not able to isolate the virus (Wölfel et al., 2020). Wastewater testing through molecular tools allows detection of the virus before cases of infection are diagnosed in the population, thereby enhancing early detection of new variants (Bonanno Ferraro et al., 2022). Thus, viral isolation, which could depend on the protocols used, is not required for wastewater surveillance. In response to the COVID-19 pandemic, during the first semester of 2020, many countries around the world implemented or strengthened the wastewater molecular surveillance of SARS-CoV-2 (Ahmed et al., 2020; Lodder and de Roda Husman, 2020; Medema et al., 2020; Nemudryi et al., 2020; Wu et al., 2022). In the Americas, the Centers for Disease Control and Prevention (CDC) implemented the National Sewage Surveillance System to strengthen surveillance capabilities for SARS-CoV-2 and other pathogens in the United States of America (Daughton, 2020). In Central America, Costa Rica also implemented SARS-CoV-2 molecular detection in sewage waters during the COVID-19 pandemic period (Barrantes et al., 2023).

This study presents the initial findings of SARS-CoV-2 detection in sewage water in Panama’s capital city and its surrounding areas, comparing these results with data obtained from the clinical genomic surveillance program. The identification of a new variant in the country through wastewater surveillance highlights the critical value of maintaining both SARS-CoV-2 genomic surveillance in clinical samples and wastewater monitoring to support a more comprehensive and integrated surveillance system.

## 2 Material and methods

### 2.1 Bioethical considerations

The wastewater genomic surveillance study has been registered in the RESEGIS platform from DIGESA from the Ministry of Health in Panamá under number 3670. The clinical molecular and genomic surveillance of SARS-CoV-2 follows the ethical requirements of Panama and was approved by the National Committee on Research Bioethics under number EC-CNBI-2020-04-46.

### 2.2 SARS-CoV-2 wastewater surveillance

#### 2.2.1 Sample collection

The wastewater genomic surveillance study was implemented in January 2024. According to the Guidelines for Environmental Surveillance for SARS-CoV-2 Detection in the Presence of a Sewage Network (World Health Organization (WHO), 2023), to capture the effluent of the denser urban populations, field visits were conducted to identify the sewerage networks of the residential areas with the highest number of people in the capital city of Panama and its surroundings for sampling. The possible circulation of SARS-CoV-2 variants in wastewater was monitored by environmental surveillance in each of the local treatment plants participating in this study in the province of Panama, the districts of 24 de Diciembre: Buena Vista and Alto del Angel, San Miguelito District: Torrijos Carter and Veranillo, and in the province of Panama Oeste in the district of Arraiján: Nuevo Arraijan and Valle Hermoso, where most of the population was located.

Wastewater samples of 50 mL were collected in duplicate in 50-mL sterile containers for each site using the random sampling method at the inlet of the treatment plants and consisted of a 24-h composition of the influent water from the Panama City sewer system without prior treatment (Rojas-Bonilla et al., 2021). All samples were collected before 9:00 a.m. to provide control for this parameter, in addition to the fact that morning hours are an accepted approach for collecting consolidated human waste using the random sampling method. Samples were stored in ice packs in an ice chest for transport. All samples were sealed and transported immediately at 2–8°C from the collection sites to the Gorgas Institute in Panama City, Panama. Once in the laboratory, the samples were conserved at 2–8°C and used for viral RNA extraction before 72 h after reception. This time between collection and analysis was selected based on previous studies to have minimal degradation of RNA from environmental samples, as it has been shown that SARS-CoV-2 RNA from intact viral particles are stable at that temperature for that time period and that viral RNA concentration decreased only 1.11% after 7 days of storage at 4°C and 11.23% after 14 days (Qiu et al., 2022; Islam et al., 2022; Torabi et al., 2023). The excess sample was frozen at −80°C. From January to June 2024, six samples per month (one per collection site) were processed for a total of 36 samples.

#### 2.2.2 Sample preparation and RNA extraction

Environmental samples were processed using the polyethylene glycol (PEG) precipitation method to concentrate virus particles. This method was previously standardized by the Universidad de la República, Uruguay (Cancela et al., 2023). Before processing, the two duplicate wastewater samples were mixed and the 45-mL composite sample was centrifuged at 4,750 × *g* for 30 min at 4°C. Then, 40 mL of the supernatant was transferred to a new tube containing 4 g of PEG 8000 MW (0.1 g/mL) and 0.9 g of NaCl (0.0225 g/mL), and the precipitate was discarded. The supernatant was gently vortexed until complete dissolution of the components and incubated for 15 min at room temperature before centrifugation at 12,000 × *g* for 2 h at 4°C. The supernatant was discarded by inversion and the pellet was centrifuged at 12,000 × *g* for 5 min and the remaining supernatant was removed. The pellet was resuspended in 200 μL of 0.01 M PBS (pH 7.4) and stored at −80°C until further extraction.

Viral RNA extraction was performed with the QIAGEN brand QIAmp^®^ Viral RNA Mini Kit (QIAGEN, Germany) according to the manufacturer’s instructions, using an internal control (IC) provided with the kit.

For human nasopharyngeal swabs, viral RNA extraction was performed with the MagMAX viral RNA extraction kit (ThermoFisher Scientific, United States) in a Thermo Scientific KingFisher flex purification system, according to the manufacturer’s instructions.

#### 2.2.3 SARS-CoV-2 RT-qPCR amplification

The COVID-19 RT-PCR Real TM Environmental kit (ATGen, Institut Pasteur de Montevideo, Universidad de la República, Uruguay) was used in the wastewater samples for *in vitro* molecular detection of SARS-CoV-2 RNA through amplification of the nucleocapsid (N) gene, with a cutoff of CT <40 in an MIC qPCR equipment (Biomolecular Systems, Australia), as described in previous studies (Cancela et al., 2023). A clinical positive sample and the synthetic RNA control from the kit were used as positive controls for this test. A multiplex RT-qPCR for Influenza virus A (M1 gene) and B (NS2 gene) and SARS-CoV-2 (N gene) was used to detect SARS-CoV-2 RNA from the clinical surveillance samples (Shu et al., 2021), using a QuantStudio 5 real-time PCR machine (ThermoFisher Scientific).

### 2.3 SARS-CoV-2 genomic surveillance from clinical and wastewater samples

SARS-CoV-2 genomic surveillance from nasopharyngeal swabs from human subjects was implemented in 2020 during the COVID-19 pandemic as described previously (Franco et al., 2021). Currently, Gorgas Memorial Institute for Health Studies (GMI) continues to receive surveillance samples for SARS-CoV-2, influenza, and respiratory viruses from all the provinces from Panama. SARS-CoV-2 genomic clinical surveillance program is part of the PAHO COVIGEN regional genomic surveillance network. SARS-CoV-2-positive clinical samples by RT-qPCR with a Ct value < 25 were selected for complete genome analysis. The selection criterion for SARS-CoV-2-positive environmental samples by RT-qPCR was a Ct value <35 for complete or partial genome analysis.

#### 2.3.1 Next-generation sequencing

First, viral RNA extracted from the wastewater samples and clinical swabs was used to generate the cDNA library for whole-genome sequencing using Illumina COVIDSEq kit, according to the manufacturer’s instructions. Simultaneously, for wastewater samples only, an RT-PCR reaction for amplification of the SARS-CoV-2 spike (S) gene was performed following the CDC protocol (CDC Protocol: LP-471,R-2D M.Wilson & M. Keller Dic 2022) to have a higher depth of coverage for this gene. Sequencing was performed using the Illumina COVIDSEq for the whole genome and Nextera XT Test for the spike gene, according to the manufacturer’s instructions and pooled using a MiSeq Reagent Kit v2 (500-cycles - MS-102-2003) on 2 × 150 cycles (MS-103-1002) paired-end runs. All sequencing data were acquired using the Illumina MiSeq sequencing platform and MiSeq Control software v2.6.2.1. The resulting reads were merged and mapped to the SARS-CoV-2 reference sequence MN908947 from Genbank using the pipeline gencomvariable, available in https://github.com/AAMCgenomics/gencomvariable. Sequences from the SARS-CoV-2 whole genomes generated from the clinical surveillance (GISAID numbers EPI_ISL_19214221, EPI_ISL_19254970, EPI_ISL_19254971, EPI_ISL_19254972, EPI_ISL_19254973, EPI_ISL_19254974, EPI_ISL_19254975, EPI_ISL_19254976, EPI_ISL_19254977, and EPI_ISL_19254978) and partial genomes generated from the wastewater surveillance (GISAID numbers EPI_ISL_19273350 spike gene, EPI_ISL_19273352, and EPI_ISL_19273353) in Panama from January to June 2024 were promptly made available in the EpiCov database in the GISAID platform (doi:10.55876/gis8.241217th).

#### 2.3.2 SARS-CoV-2 variant characterization

Sequences from the SARS-CoV-2 complete genomes and partial genomes were classified using Pangolin and Nextclade algorithms (Aksamentov et al., 2021).

## 3 Results

### 3.1 SARS-CoV-2 detection through wastewater and human clinical surveillance

**Figure 1:**
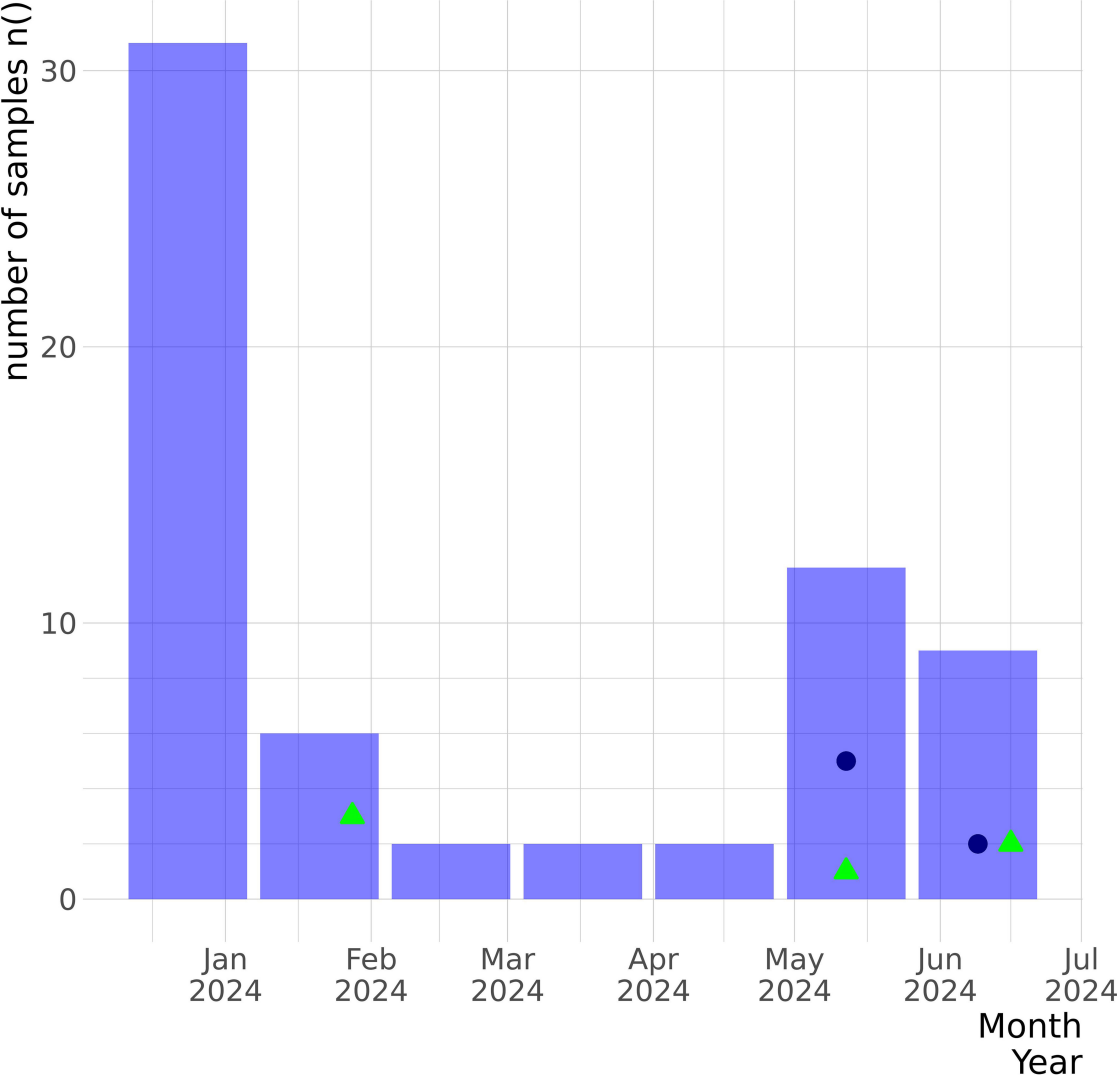
Epidemiological graph of SARS-CoV-2 samples detected by the GMI in Panama between January and June 2024 from the human clinical surveillance and the wastewater surveillance programs. The number of confirmed SARS-CoV-2-positive clinical swabs received by GMI for molecular surveillance from the national febrile human surveillance program for SARS-CoV-2, influenza, and respiratory viruses during this period is represented by the purple bars. The SARS-CoV-2-positive clinical cases that were sequenced are represented by the blue navy dots. Through the wastewater surveillance program implemented in January 2024, six samples were received monthly for molecular testing, and SARS-CoV-2-positive samples are represented by green triangles. The *y*-axis represents the number of samples, and the *x*-axis denotes the collection dates.

From the 36 environmental samples collected from 1 January to 30 June 2024 for the wastewater surveillance, 6 (16.67%) were positive for SARS-CoV-2 genetic material: 3 positive samples from January, 1 from May, and 2 from June (**Figure 1**). Through human SARS-CoV-2, influenza, and respiratory virus surveillance, we received 822 suspected clinical swab samples during the same period from all the national territories, of which 65 (7.91%) were positive for SARS-CoV-2 by RT-qPCR (**Figure 1**).

All SARS-CoV-2-positive samples detected through the wastewater surveillance met the selection criteria for sequencing (CT < 35); however, we obtained one sequence of the spike gene (EPI_ISL_19273350) and two partial genomes (average size of 91.1% of bp, median reads 154,170 with a median coverage of 88%) (**Table 1**). From the human surveillance program, of the 65 SARS-CoV-2-positive clinical swabs, only 10 met the criteria for sequencing (CT < 25) and were sequenced to obtain their complete genomes (median reads 274,244 with a median coverage of 99.57%).

**Table 1:** Epidemiological information of SARS-CoV-2-positive cases detected through molecular and genomic wastewater surveillance.

Collection site: Neighborhood (district)	Collection date	Results: positive RT-qPCR Ct values (variant)
Buena Vista (24 de Diciembre)	31 January 2024	33.7
Alto de Ángel (24 de Diciembre)	31 January 2024	31.22
Nuevo Arraiján (Arraiján)	31 January 2024	33.7
Veranillo (San Miguelito)	13 May 2024	34.91 (JN.1.16.1)
Buena Vista (24 de Diciembre)	20 June 2024	27.5 (JN.1.16.1)
Valle Hermoso (Arraiján)	20 June 2024	29.09 (JN.1.16.1)

### 3.2 Different SARS-CoV-2 variants identified by wastewater and human surveillance

**Figure 2:**
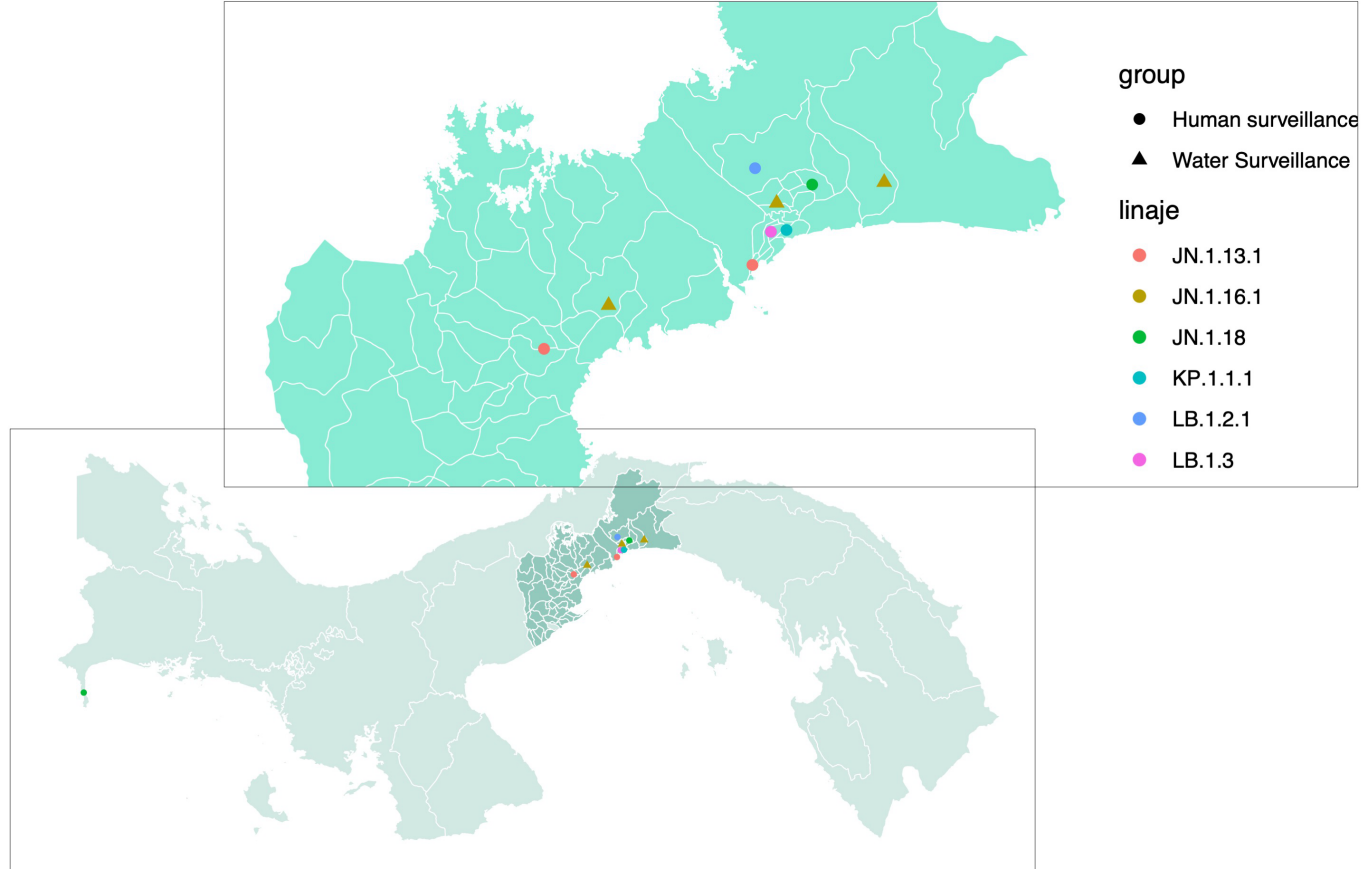
Map of SARS-CoV-2 variants detected through human clinical and wastewater genomic surveillance programs. Map of Panama zooming in on the provinces of Panama and Panama Oeste, showing SARS-CoV-2 variants (color-coded: pink, JN.1.13.1; maroon, JN.1.16.1; green, JN.1.18; aquamarine, KP.1.1.1; blue, LB.1.2.1; lilac, LB.1.3) identified by the human surveillance (dots) from human-positive clinical samples and by the wastewater monthly surveillance (triangles).

SARS-CoV-2 partial genome sequences obtained from wastewater surveillance were used to determine which variants were circulating in Panama during May and June 2024. The three sequences obtained showed the presence of the SARS-CoV-2 Omicron sublineage JN.1.16.1 (**Table 1**) in all the analyzed districts from Panama province (24 de Diciembre and San Miguelito) and Panama Oeste province (Arraiján) (**Table 1**; **Figure 2**). On the other hand, SARS-CoV-2 molecular and genomic surveillance from human samples detected five other Omicron sublineages (JN.1.13.1, JN1.18, KP.1.1.1, LB.1.2.1, and LB.1.3), but not JN.1.16.1 (**Figure 2**). The highest diversity of sublineages was observed in the capital city of Panama in Panama province, whereas in Panama Oeste province, only the JN.1.13.1 sublineage was detected. These other Omicron sublineages were detected from human cases from the same districts where JN.1.16.1 was detected by the wastewater surveillance, but not the same neighborhoods.

## 4 Discussion

Because of Panama’s role as a human transit hub through its international airports and ports, as well as a route for illegal migration to the United States of America from around the world (Higuita et al., 2022), a wastewater surveillance program for early detection and monitoring of any pathogen of public health concern would have a strong benefit for public health measures not only at the national level, but also at the regional and global level (Hill et al., 2024).

Here, we describe the successful implementation of SARS-CoV-2 molecular and genomic surveillance in the wastewater of the capital city and its surroundings in Panama highlighting the detection of a notable variant during the initial 6 months of the program. The omicron sublineage JN.1.16.1 was identified for the first time in the country through this surveillance in the most densely populated regions, as the two provinces analyzed represent more than 50% of the country’s population. Interestingly, this sublineage was not detected through the surveillance of clinical human samples.

Of the 36 samples analyzed from the wastewater surveillance, 6 (16.67%) were positive, whereas the percentage of positivity for the samples obtained from the human molecular surveillance was 7.91% (65/822). The discrepancy may be attributed to the nature of the samples. Wastewater captures viruses shed by a population, including those who continue shedding virus for days after recovery (Chen et al., 2020; Xu et al., 2020). In contrast, detecting the virus in nasopharyngeal swabs from patients depends on factors such as the timing of symptom onset and the precision of sample collection techniques (Ganyani et al., 2020; Kucirka et al., 2020; Wölfel et al., 2020).

Moreover, there are differences in the samples’ time of transportation between both surveillance. The wastewater samples arrived at GMI in less than 6 h after collection; while the samples collected for clinical surveillance could arrive 72 h after collection, once at the GMI laboratory, both types of samples are conserved at 4°C and viral RNA was extracted before 72 h (maximum time limit, average was 48 h after arrival at the laboratory). It has been reported that in environmental samples, free viral RNA could be degraded after 48 h even when stored at 4°C, but at this cold temperature, RNA from intact SARS-CoV-2 virus particles could be preserved for periods of time longer than 72 h (Qiu et al., 2022; Islam et al., 2022; Torabi et al., 2023), which is the maximum time of analysis of wastewater samples in our study. This suggests that RNA degradation in our environmental samples should be minimal, which is also supported by the fact that the percentage of SARS-CoV-2-positive samples from the wastewater surveillance (16.67%) is higher than from the clinical swabs (7.91%). Finally, the RT-qPCR methods used for each surveillance are different and clinical surveillance uses the methodology recommended by the CDC (Shu et al., 2021); on the other hand, the methodology used for wastewater surveillance was developed for this specific purpose by the Pasteur Institute and the Universidad de la República in Uruguay (Cancela et al., 2023), and the sensitivity of each method could be different. The CDC assay detects five copies of SARS-CoV-2 RNA per reaction (Shu et al., 2021); however, the limit of detection (LoD) of the environmental kit has not been described, but it has been optimized for this type of specimen (Cancela et al., 2023).

Similarly, there is a notable difference in the percentage of positive samples that underwent sequencing between the two surveillance systems. During the analyzed period, 50% of SARS-CoV-2-positive samples from wastewater surveillance were sequenced, compared to only 15.39% from human surveillance. This disparity arises from stricter selection criteria in the latter case, where a CT value of <25 is required to have complete genome sequences with high depth, whereas a CT value of <35 suffices for wastewater surveillance. These criteria align with distinct objectives: wastewater surveillance aims to obtain a partial genome for variant and sublineage identification, whereas human clinical surveillance strives for complete genome sequencing to facilitate deep phylogenetic analysis. This difference in percentage of positive and sequenced compared to collected samples suggests that the environmental surveillance has a better cost–benefit ratio (Daughton, 2020). Moreover, the surveillance based on sewage water has a communitary focus with less ethical issues as samples are anonymous (Hill et al., 2024; Smyth et al., 2022).

We detected five different Omicron sublineages in the clinical swabs collected between May and June 2024. JN.1.13.1 was detected in both provinces (Panama and West Panama), while Panama City had the highest diversity with the detection of these five sublineages in human cases from different neighborhoods. On the other hand, only sublineage JN.1.16.1 was detected in both provinces through wastewater surveillance. This difference in the SARS-CoV-2 sublineages identified by each surveillance system could be explained by the surveillance system itself, as wastewater allows the identification of the main viruses circulating in a population group, whereas clinical surveillance depends on people with symptoms assisting the healthcare system, which could include a bias for symptom-inducing variants (Fan et al., 2022; Shang et al., 2022), a hypothesis that should be tested in future studies. Moreover, in this study, we did not take into account the vaccine status of the population of each province, and similarly, this information was not available from the patients detected through the clinical surveillance. Thus, vaccination could also impact the sublineage detected for each surveillance. This difference could also be explained by the fact that clinical samples and wastewater samples were not collected in the same neighborhoods, but the fact that JN.1.16.1 was detected in wastewater from three different neighborhoods in both provinces suggests that this sublineage is circulating throughout the city and its surroundings. Finally, clinical samples were collected throughout the period analyzed, with variations in the number of samples collected per day depending on the number of ILI in the population, while wastewater samples were collected once a month, allowing the analysis of the virus circulating that day. Nevertheless, JN.1.16.1 was identified in samples collected in May and June. Regardless of the differences between the two surveillance programs, the presence of JN.1.16.1 in the sewage system suggests a cryptic transmission of this sublineage in the two most populous provinces of the country that was not detected by the human clinical genomic surveillance system. Another reason explaining why this sublineage has not been detected by the clinical genomic surveillance may be the low number of positive clinical swabs analyzed for sequencing in a post-vaccination period, when most SARS-CoV-2 cases are likely to be asymptomatic or mild (Antonelli et al., 2023; North et al., 2022) and are not included in the ILI surveillance. SARS-CoV-2 cryptic transmission has been previously reported, highlighting the importance of WW surveillance (Smyth et al., 2022). As seroprevalence studies have shown that a small percentage of domestic and urban small mammals have been in contact with SARS-CoV-2 (Tinto et al., 2023; Klestova, 2023), there is always a possibility that part of the SARS-CoV-2 detected in the wastewater system comes from other mammals cohabitating in the city. However, one of the limitations of this study is that SARS-CoV-2 surveillance currently does not include animals and future studies are needed to determine their possible role in SARS-CoV-2 cryptic transmission in urban settings.

While the wastewater surveillance system has been successfully implemented, there are many areas for improvement. Increasing the frequency of sampling could improve sensitivity, enabling earlier detection of introduction or rise of specific SARS-CoV-2 variants. In addition, the wastewater surveillance program should be expanded to include the detection and monitoring of other viruses and pathogens, such as hepatitis A, hepatitis E, seasonal respiratory viruses, enteroviruses, and polioviruses, as practiced in many countries. Currently, molecular and genomic surveillance for SARS-CoV-2 and other pathogens is concentrated in the capital and its surrounding areas; thus, to strengthen the system, it is essential to expand these surveillance efforts by incorporating additional collection points across the national sewage network.

Panama is a country with only 4,395,00 inhabitants, with 35.4% living in the capital city of Panama^1^, where the main international airport and cruise port are located where this study was conducted. Through the Tocumen International Airport, Panama has received 18,026,533 passengers in 2023 with an increase of 9.2% in the first 6 months of 2024^2^. Exchanges are also increased with the Panama Canal and cruise ports; Panama received 574,000 international visitors between January and February 2024^3^. Moreover, Panama is a route for illegal migration to the United States of America from around the world, with more than 520,000 migrants crossing the Darien gap in 2023^4^, with an impact in public health (Higuita et al., 2022). Owing to Panama’s role as a human transit hub, strategic points at airports, ports, and land borders should be included in the wastewater surveillance program for early detection and monitoring of any pathogen of public health concern.

## 5 Conclusion

In conclusion, the implementation of a genomic surveillance for SARS-CoV-2 in wastewater in the metropolitan area of Panama’s capital city has proven to be an invaluable tool. Within the first 6 months of its application, it enabled the detection of a SARS-CoV-2 sublineage not identified by clinical surveillance, highlighting the complementary nature of both programs. Wastewater surveillance through molecular detection has demonstrated its efficiency in tracking viral trends within specific geographic areas.

The integration of wastewater surveillance with clinical surveillance represents a significant advancement for epidemiological programs in Panama and the region. Moreover, when combined with genomic analysis, this approach could serve as a sentinel surveillance system, providing early warning for future potential outbreaks. A comprehensive surveillance program also holds the potential to predict the number of cases through predictive modeling, offering crucial insights into the environmental and temporal variation of viruses such as SARS-CoV-2.

## Data Availability

The datasets presented in this study can be found in online repositories. The names of the repository/repositories and accession number(s) can be found in the article/supplementary material.

## Footnotes

1 Instituto Nacional de Estadística y Censo, Contraloría de la República, https://www.inec.gob.pa.

2 Tocumen international airport, https://logistics.gatech.pa/plataforma-logistica/activos-logisticos/aeropuertos/aeropuerto-internacional-de-tocumen/.

3 Autoridad de Turismo de Panamá, https://www.atp.gob.pa.

4
https://www.thenewhumanitarian.org/maps-and-graphics/2024/01/15/darien-gap-migration-crisis-six-graphs-and-one-map#:~:text=A%20record%20520%2C000%20migrants%20crossed,surge%20is%20expected%20in%202024.
